# Wavelength shift strategy to enhance lipid productivity of *Nannochloropsis gaditana*

**DOI:** 10.1186/s13068-018-1067-2

**Published:** 2018-03-19

**Authors:** Min-Gyu Sung, Jong-In Han, Bongsoo Lee, Yong Keun Chang

**Affiliations:** 1 Department of Chemical and Biomolecular Engineering, KAIST, 291 Daehak-ro, Yuseong-gu, Daejeon, 305-701 Republic of Korea; 2 Department of Civil and Environmental Engineering, KAIST, 291 Daehak-ro, Yuseong-gu, Daejeon, 305-701 Republic of Korea; 3 Advanced Biomass R&D Center, #2502 Building W1-3, 291 Daehak-ro, Yuseong-gu, Daejeon, 305-701 Republic of Korea

**Keywords:** Microalgae, Fluorescent dye, Wavelength, Biofuel

## Abstract

**Background:**

Microalgae, being a phototroph, grow in the presence of light, and utilizing photons in narrow and specific range of wavelengths. There have been numerous attempts to take advantage of this trait of wavelength-dependent growth for the purpose of increasing biomass productivity. One potential option involves wavelength conversion of sunlight. In the present study, three fluorescent dyes with blue, red, and green emission spectra were employed with the aim of improving sunlight utilization efficiency and thus enhancing biomass and lipid productivity of *Nannochloropsis gaditana*.

**Results:**

When DPA and R101 were used to enrich blue and red spectra, biomass productivity of *Nannochloropsis gaditana* was increased by 35.1 and 40.3%, respectively. The maximum quantum yield values were higher than 0.6 at the early stage of growth for the cultures grown under DPA- and R101-modified solar radiation. Chlorophyll *a* content was also 57.0 and 32.3% higher than the control at the early growth stage under DPA- and R101-modified solar radiation, respectively. This stimulation of photosynthetic activity at the early growth stage correlated well with rapid growth under DPA- and R101-modified light during the first 4 days of cultivation. Lipid productivity consequently increased by 26.9 (DPA) and 39.4% (R101) after 10 days of cultivation. An immediate effect on lipid induction was observed in cultures under modified light, which exhibited 19.1% improvement in lipid content at the cost of some degree of impaired growth.

**Conclusion:**

Fluorescent dyes with the capability of enriching wavelengths of light favored by the algal photosystem could indeed be an effective means of promoting growth of *Nannochloropsis gaditana*. This strategy would be particularly powerful for mass cultivation where sunlight is the only economically viable option for illumination.

**Electronic supplementary material:**

The online version of this article (10.1186/s13068-018-1067-2) contains supplementary material, which is available to authorized users.

## Background

Microalgae are phototropic organisms that grow using sunlight as an energy source and water as an electron source. This, along with the ability to use CO_2_ as a carbon source, makes them an ideal candidate for an eco-friendly and renewable feedstock for the production of the next generation of chemicals and fuels [1]. Microalgae are equipped with the same photosynthetic apparatus as all other phototrophs. Through the light harvesting system, light energy is captured and converted to chemical energy in the forms of ATP and NADPH, which power the ensuing biosynthesis of molecules such as bio-oils [2, 3]. To make microalgae-derived bioproducts such as biofuels economically viable, however, further advances in genetic engineering or innovation of cultivation technology are necessary.

The trait of growing under light, which is among the most important advantages of microalgae, poses challenges when it comes to mass production. For example, when microalgae are subjected to excessive light intensity, the culture may die due to photoinhibition; if the light intensity is too low, the growth becomes suboptimal [4]. In addition to the intensity, parameters such as frequency and wavelength are also important, and especially so when artificial light sources are employed [5]. When microalgae grow with natural light containing the full spectrum of solar radiation, they absorb and make use of only the wavelength within the visible range of 400–700 nm, which is known as photosynthetic active radiation (PAR) [6]. Ultraviolet (UV) and far-red ranges are not only unused but also harmful [7, 8]. Even within the PAR, there are different preferred colors among algae types on account of varied compositions of pigments in the light harvesting complex. *Chlorella* spp., for example, has chlorophyll *a* and *b* as major pigments, which take up blue and red photons and not green ones. On the other hand, *Nannochloropsis* spp. lacks chlorophyll *b* [9] and instead possesses xanthophyll carotenoids such as violaxanthin, zeaxanthin, and vaucheriaxanthin. This allows them to additionally utilize blue–green wavelength.

There have been some attempts to take advantage of this wavelength-dependent property for the purpose of increasing biomass productivity. Light-emitting diodes (LEDs), which emit only a narrow range of photons, are a light source well-suited for this purpose. Schulze et al. found that the illumination of blue- and red-colored LEDs, the wavelengths preferred by the microalgae, led to a substantial increase in growth [10]. Okumura et al. made a similar observation that *Nannochloropsis* appeared to be particularly responsive to these wavelengths in that lipid content as well as growth was affected [11]. The use of LEDs, though evidently convenient and beneficial, is limited to the indoor production of functional, high value bio-products such as medicine. Exceedingly high electricity costs prohibit their application to fuels and commodity products. For practical purposes, a sunlight-based approach with wavelength conversion can be a potential option. This can be realized by means of a light filter or fluorescent dyes; the latter is readily available at a relatively low price, and very low concentration is required to achieve desired level of solar spectrum modification [12, 13].

Fluorescent dyes are special chemical compounds typically with aromatic rings, and they are capable of absorbing photons of a certain range of wavelength of incident light and subsequently emitting photons with longer wavelength. These can be used to convert the unused parts of the solar spectrum into a usable blue or red color, thereby enhancing the growth of microalgae and lipid productivity. For instance, Detweiler et al. reported that an intentional increase in both blue and red wavelengths resulted in growth improvement in four different species [14]. However, this rather straightforward and advantageous strategy does not always produce desired outcomes: e.g., Mohsenpour et al. observed that spectral conversion resulted in impairment of growth in *Chlorella vulgaris* [15]. Therefore, it appears that there are certain controlling factors that affect the performance of microalgae in utilizing various wavelengths of light.

In an effort to unravel them, we selected three commercial fluorescent dyes, namely, 9,10-diphenylanthracene (DPA), rhodamine 101 (R101), and rhodamine 110 (R110), and examined their functions and potential for the cultivation of *Nannochloropsis gaditana* in a flat-panel photobioreactor (PBR).

## Results

### Spectral conversion of incident sunlight using fluorescent dyes

**Fig. 1:**
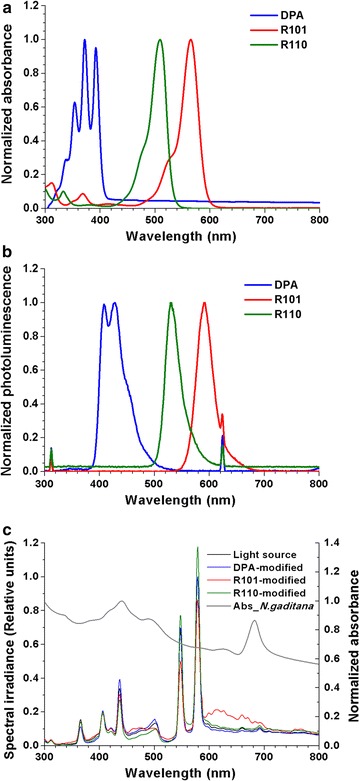
Spectral characteristics of fluorescent dyes. Spectra of absorption (**a**), emission (**b**) and converted spectral irradiance (**c**). Absorption spectrum of *N. gaditana* was also presented in (**c**)

Three different fluorescent dyes were used to convert the spectrum of incident sunlight. To measure the absorption and emission characteristics, all three were dissolved in ethanol at concentration of 10^−5^ M. The entire spectrum from 300 to 800 nm was checked and normalized to each peak wavelength (Fig. 1). UV was converted to the visible range using 9,10-diphenylanthracene (DPA). As shown in Fig. 1, it is capable of absorbing photons in a range of 300–400 nm (peak at 370 nm) and emitting blue photons with a wavelength range of 400–500 nm (peak at 425 nm). Rhodamine 110 (R110) absorbed light at 420–550 nm (peak wavelength at 509 nm) and emitted 500–620 nm (peak wavelength at 529 nm) (blue to green). Finally, rhodamine 101 (R101) mainly absorbed 460–620 nm of wavelength (peak wavelength at 565 nm) and emitted 550–700 nm (peak wavelength at 586 nm) (green to red).

Based on the absorption and emission characteristics of each dye, the light spectra for each condition were found to have been changed (Fig. 1c). It was shown that the overall conversion of the light source was influenced by both absorption and emission range of a dye. When DPA was used, the proportion of blue light range (400–500 nm) increased with corresponding decrease of the UV range (300–400 nm). As for R110, the proportion of blue light (400–500 nm) was reduced, while the irradiance peaks at the green range (500–620 nm) increased. Lastly, when R101 was used to modify light source, green light (500–620 nm) was reduced and red (550–700 nm) has increased substantially.

### Effect of wavelength shift on growth

**Fig. 2:**
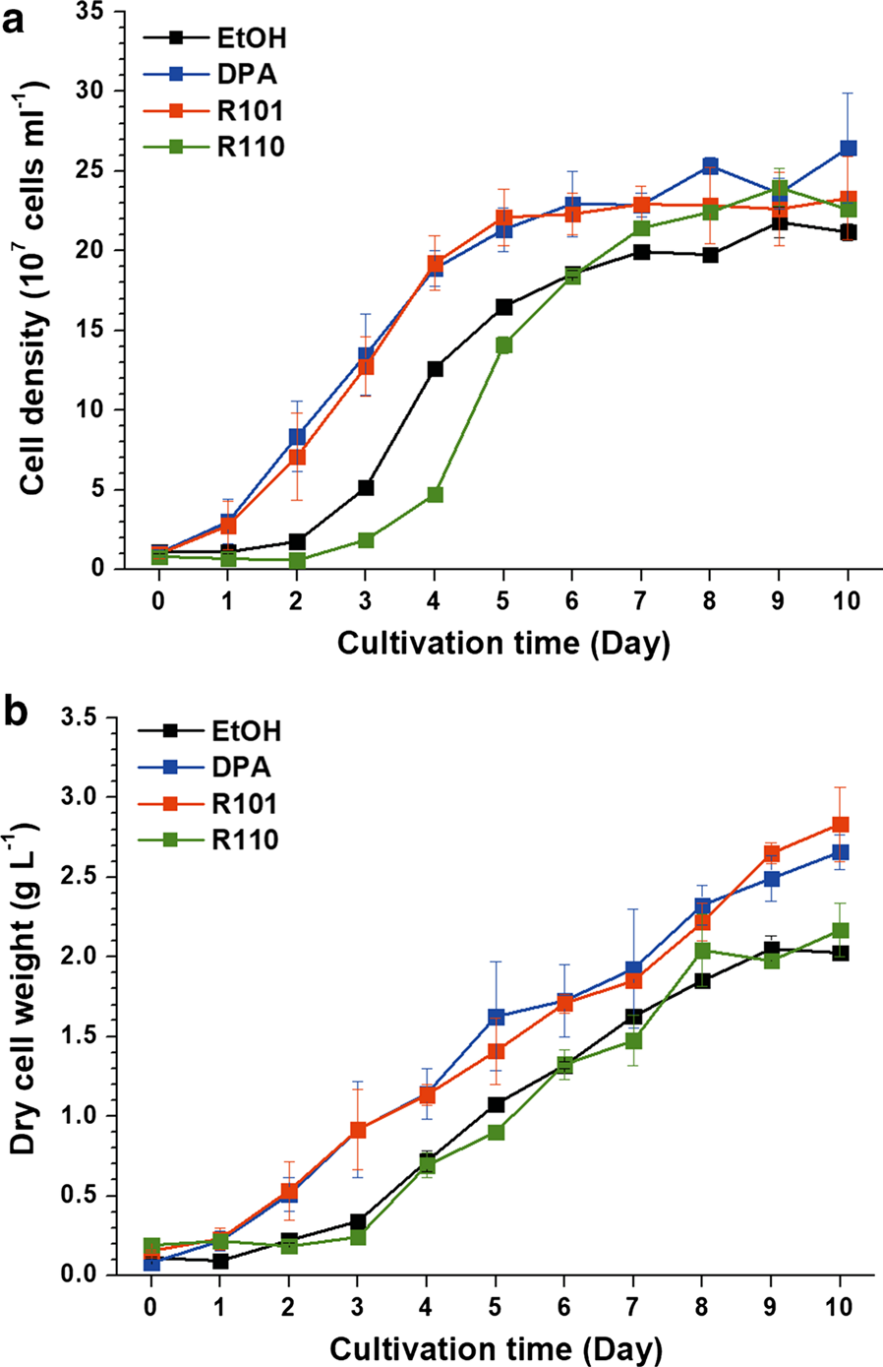
Growth of *N. gaditana* with fluorescent dyes. Growth curves based on cell density (**a**) and dry cell weight (**b**). Data points represent means and standard errors (*n* = 3)

The growth of *N. gaditana* was monitored under batch cultivation mode with the three dyes for 10 days (Fig. 2). The control experiment used ethanol without any dye. The effects on growth were clearly seen in terms of both cell number and dry weight. This showed that the usage of DPA and R101 resulted in the promotion of cell division and biomass production. The maximal specific growth rate (*μ*_max_) with DPA-modified light was 0.716 day^−1^, with a final biomass of 2.66 g L^−1^, 31.3% higher than with the ethanol control (Table 1).

**Table 1:** Maximum specific growth rate and final biomass of *N. gaditana* with different dye materials

Experiment	EtOH	DPA	R101	R110
*μ*_max_ (day^−1^)	0.654 ± 0.123	0.716 ± 0.119	0.686 ± 0.052	0.660 ± 0.105
Final biomass (g L^−1^)	2.025 ± 0.020	2.658 ± 0.112	2.833 ± 0.207*	2.167 ± 0.151

The data points represent the average of samples and error bars indicate standard error (*n* = 3). Significant differences, as determined by Student’s *t* test, are indicated by asterisks (* *P* < 0.05)

Enriched red light via R101 also had a substantially positive impact on the growth of *N. gaditana*. The maximal growth rate with R101-modified light was found to be 0.686 day^−1^ (Table 1). The final biomass for 10 days was increased by 39.9% up to 2.83 g L^−1^, which was the highest value obtained under all conditions. On the other hand, R110 exhibited a different pattern. Compared to the other conditions, the growth rate lagged behind for the first 3 days of exposure to the R110-modified light, and the growth rate was even lower compared to the control (Fig. 2). After the initial lag period, however, the cells started to grow at a similar rate compared to the others (Table 1), implying that the microalgae required an adaptation period. The final biomass density was comparatively low (2.17 g L^−1^), primarily due to the extended lag phase. It appears that the enrichment of green color has a negative effect.

### Changes of photosynthesis with wavelength shift

**Fig. 3:**
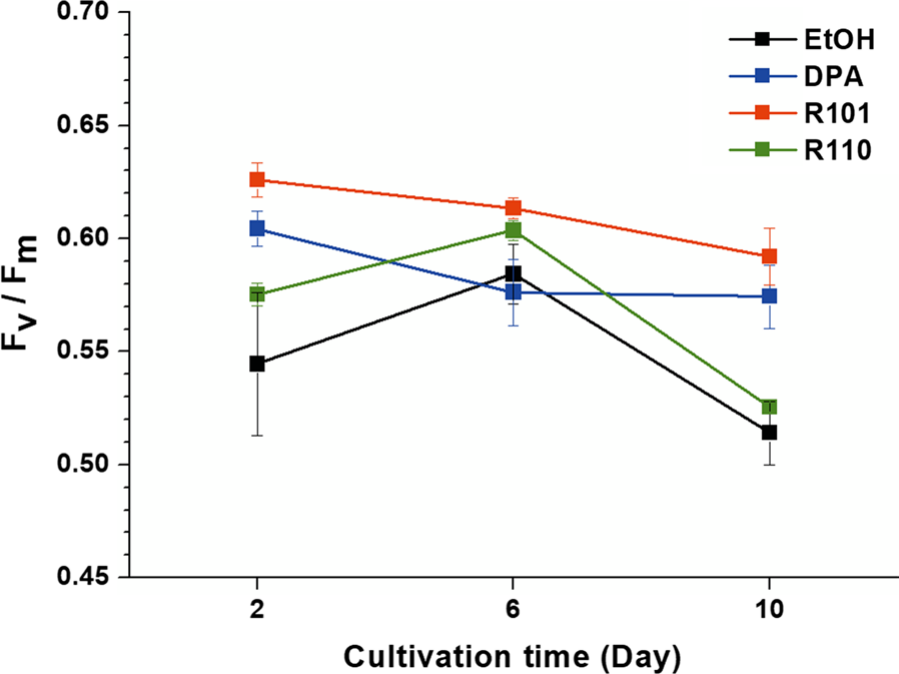
Maximal quantum yields of *N. gaditana*. All measurements were achieved at 440 nm. Data points represent means and standard errors (*n* = 3)

To elucidate the possible mechanisms behind the varied responses in connection with photosynthesis, the maximal quantum yield (*F*_v_/*F*_m_) values of PS II were estimated using a multi-color-PAM for each light condition with different fluorescent dyes (Fig. 3). Samples from 2/6/10 days, each of which belonged to early-exponential/late exponential/stationary growth phases, were analyzed.

At the early stage of growth, the *F*_v_/*F*_m_ value was significantly higher in cells grown under DPA and R101 modified light (Fig. 3). In both cases, *F*_v_/*F*_m_ was higher than 0.6. For cell growth under R110-modified light, *F*_v_/*F*_m_ was as low as the control case, at 0.575. This result was well-reflected by the growth pattern in the early stage of cultivation up to day 3, when rapid cell division took place (Fig. 2).

Among cultures at day 6, the highest *F*_v_/*F*_m_ value was found in the culture grown under R101-modified light, while the others remained mostly unchanged (Fig. 3). At this point of cultivation, cells grown under R101- and DPA-modified light were near the stationary phase, but cells were still in the active growth stage under R110- and the ethanol-modified light (Fig. 2). The *F*_v_/*F*_m_ values of DPA- and R101-modified light dropped to 0.576 and 0.616, respectively; both exhibited lower *F*_v_/*F*_m_ compared to day 2. On the other hand, the R110-modified culture and the control were found to have an increased *F*_v_/*F*_m_ value compared to day 2. This caused the discrepancy among dyes to become narrower.

*F*_v_/*F*_m_ values at day 10, corresponding to the time frame in which *N. gaditana* was in the stationary phase, again diverged (Fig. 3). Due to being in the late stage of growth, the cells were expected to display reduced *F*_v_/*F*_m_ values, which was the case in general. This was especially true for the R110 and ethanol control. *F*_v_/*F*_m_ was 0.526 for cultures under R110-modified light and 0.514 for the control, which were indeed substantially lower compared to normal values of *Nannochloropsis* [16]. DPA and R101, on the other hand, behaved differently. For these two, the *F*_v_/*F*_m_ values remained at around 0.6, which does not coincide well with the cell density (Fig. 2a), because cell division ceased even from day 6 of cultivation.

**Fig. 4:**
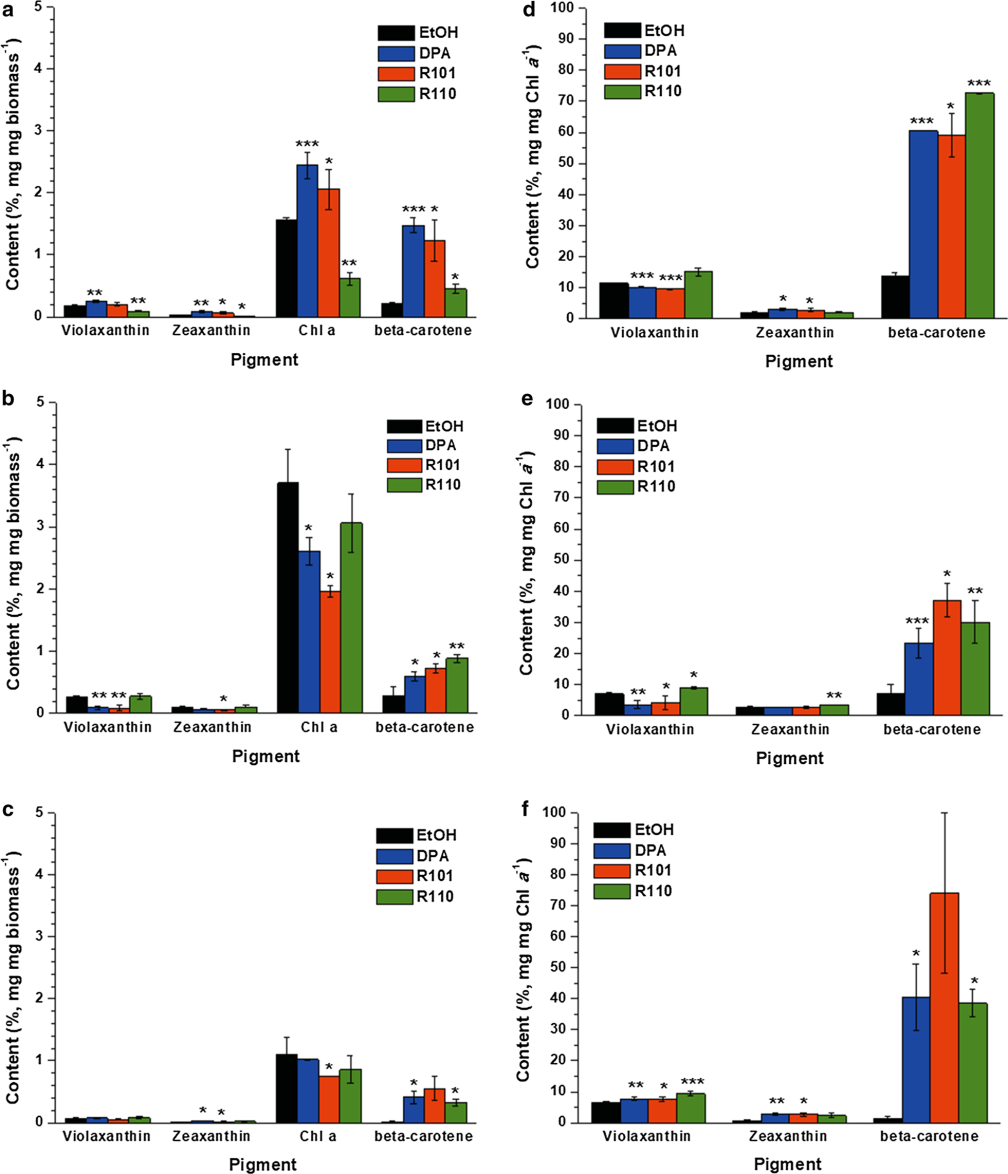
Pigment compositions along with cultivation time. Day 2 (**a**, **d**), day 6 (**b**, **e**), day 10 (**c**, **f**). The data points represent the average of samples and error bars indicate standard error (*n* = 3). Significant differences, as determined by Student’s *t* test, are indicated by asterisks (*P < 0.05, **P < 0.01, ***P < 0.001)

*Nannochloropsis* is known to possess chl *a* as a sole component of the chlorophyll family, also in addition to beta-carotene and several xanthophyll pigments [9]. Any compositional change of the pigments was monitored according to the converted wavelength of incident solar irradiation (Fig. 4). In every experimental condition, chl *a* was indeed the predominant component among the total pigments. Beta-carotene, the second most abundant pigment, showed significant variations with respect to the experimental conditions. At day 2 and day 10 of cultivation, beta-carotene content was elevated up to over 30% of the level of chl *a*, while the portion was highly reduced at day 6, which corresponds to the late exponential growth phase.

At the early stage of growth, DPA caused the amount of chl *a* to increase dramatically to 2.44% of the total biomass compared to the control case (1.56%). A similar stimulation was also found for R101, where chl *a* increased by 32.3% compared to the control. This correlates well with the high *F*_v_/*F*_m_ values found in two experimental conditions (Fig. 3). The results were somewhat opposite for the cells grown under R110-modified light (green), which exhibited a reduction of chl *a* content down to 0.62% of biomass, 2.5-fold lower than the control. Instead, violaxanthin and beta-carotene, which are accessory pigments in *N. gaditana*, proportionally increased when R110 was used for cultivation.

The compositional change was also observed at the 6th day of cultivation (Fig. 4b, e). For DPA- and R101-modified light, chl *a* content remained similar to that at day 2, which was 2.61 and 1.96% of biomass, respectively. R110-modified light and the ethanol control exhibited significant increases in chl *a* content, from less than 2% of biomass at day 2 to 3.70 and 3.06% at day 6, indicating that photosynthesis was more active at this point of cultivation. The overall tendency coincided almost exactly with the observed *F*_v_/*F*_m_ values (Fig. 3).

At the end stage of cultivation (day 10), *N. gaditana* had a very different composition (Fig. 4c, f). Chl *a* content markedly decreased for all the cases (around 1% of biomass), whereas accessory pigments including beta-carotene took up a greater portion (about 0.4%). The only exception to this was the control, wherein the accessory pigment consisted of 0.02% of biomass. As for violaxanthin, its content within the cells was roughly 7% of chl *a* for all the conditions.

### Effect of wavelength shift on lipid productivity

**Fig. 5:**
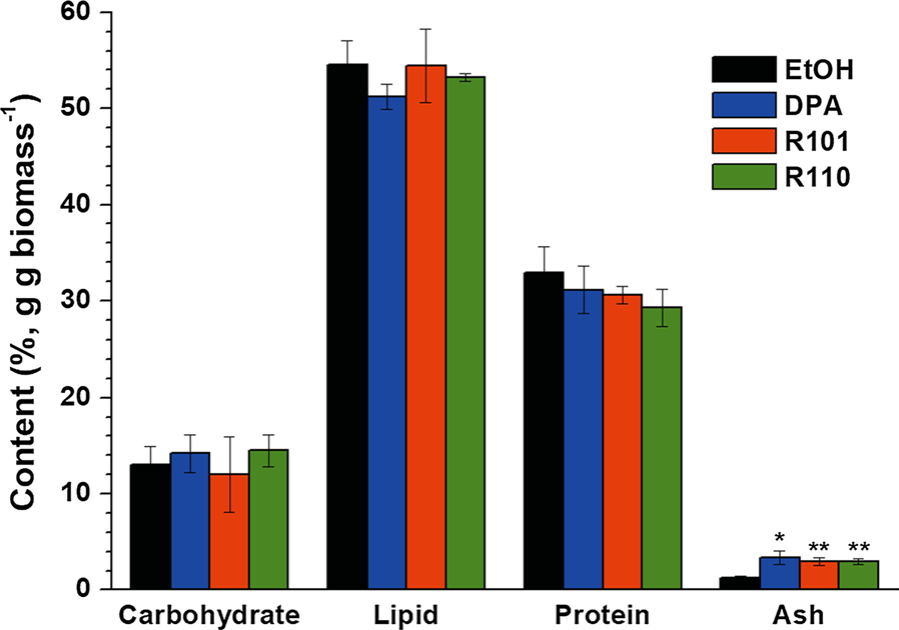
Biochemical composition of *N. gaditana* at different incident wavelength conditions after 10 days of cultivation. The data points represent the average of samples and error bars indicate standard error (*n* = 3). Significant differences, as determined by Student’s *t* test, are indicated by asterisks (*P < 0.05, **P < 0.01, ***P < 0.001)

The biochemical composition of *N. gaditana* was examined at the end of cultivation (Fig. 5). The cells entered the stationary phase in all cases and accumulated a large quantity of storage materials (e.g., lipids), primarily due to nitrogen deprivation. Total lipid contents were over 50% of dry biomass for all the conditions including the control, while the carbohydrates and proteins were below 50%.

The overall biomass and lipid productivities were estimated and are listed in Table 2. *N. gaditana* grown under modified light showed varied results in lipid productivity. Lipid productivity was higher for cultures grown under DPA-(0.132 g L^−1^ day^−1^) and R101-modified light (0.145 g L^−1^ day^−1^) in comparison with the control. R110-modified light resulted in a reduction of the lipid productivity down to 0.105 g L^−1^ day^−1^. For DPA and R101, the biomass productivity increased by 35.1 and 40.3%, respectively.

**Table 2:** Total lipid content and biomass/lipid productivities of *N. gaditana* at each wavelength condition

Experiment	EtOH	DPA	R101	R110
Lipid content (%, g g biomass^−1^)	54.5 ± 2.5	51.2 ± 1.3	54.4 ± 3.8	53.2 ± 0.4
Biomass productivity (g L^−1^ day^−1^)	0.191 ± 0.001	0.258 ± 0.013*	0.268 ± 0.023*	0.198 ± 0.011
Lipid productivity (g L^−1^ day^−1^)	0.104 ± 0.005	0.132 ± 0.007**	0.145 ± 0.011**	0.105 ± 0.006

The data points represent the average of samples and error bars indicate standard error (*n* = 3). Significant differences, as determined by Student’s *t* test, are indicated by asterisks (* *P* < 0.05, ** *P* < 0.01)

**Fig. 6:**
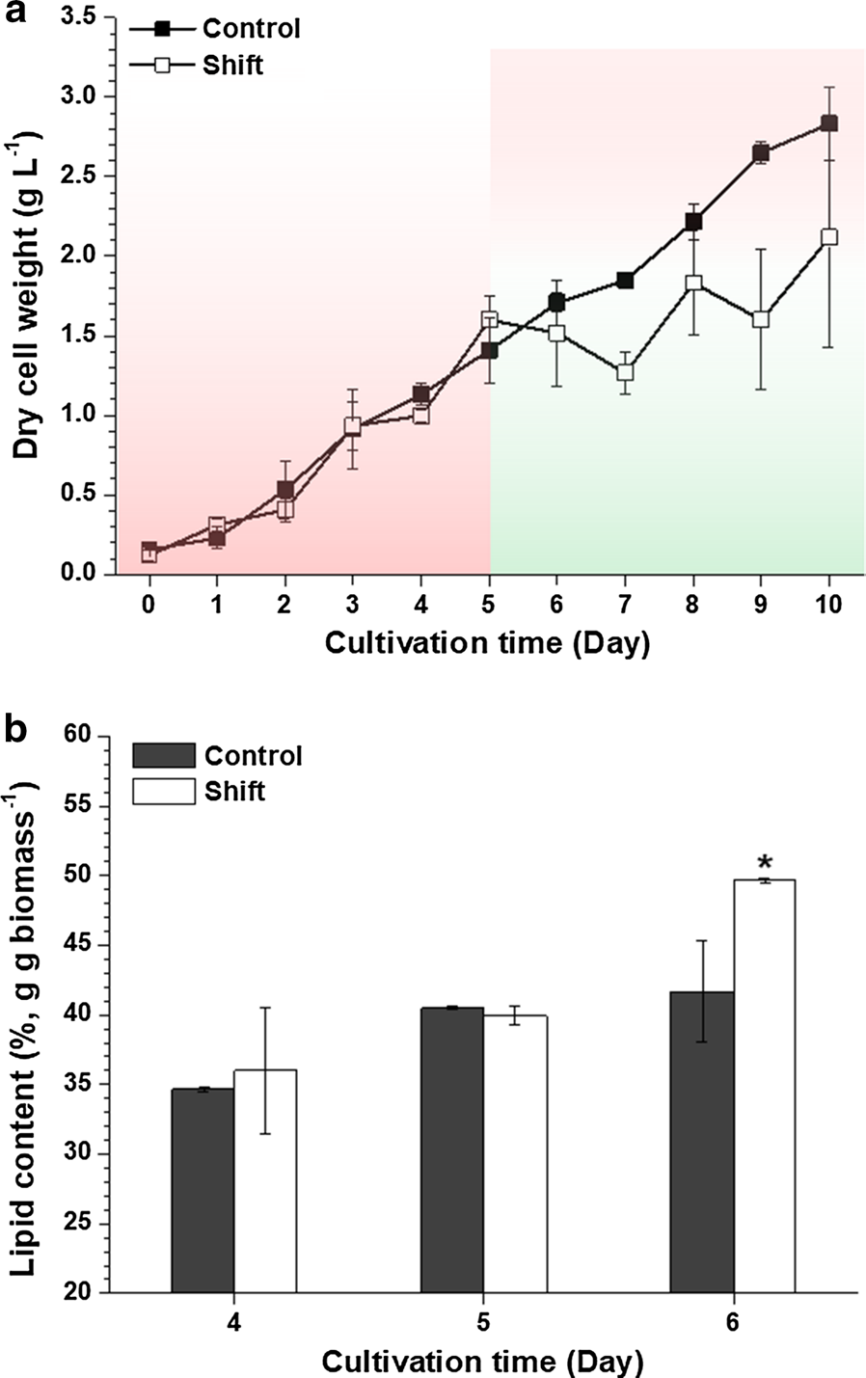
Change of growth and lipid content in *N. gaditana* with switching from R101 to R110. Growth profile (**a**) and lipid content (**b**). Control indicates dye maintained with R101, and shift indicates dye having shifted at day 5. The data points represent the average of samples and error bars indicate standard error (*n* = 3). Significant differences, as determined by Student’s *t* test, are indicated by asterisks (*P < 0.05, **P < 0.01, ***P < 0.001)

To see how modifying the wavelength composition of incident light affected lipid productivity, *N. gaditana* cells were first exposed to R101-modified light for the first 5 days before being switched to R110-modified light (Fig. 6). While *N. gaditana* grew exponentially under R101-modified light, the growth ceased almost immediately when the culture was switched to R110-modified light, likely due to sudden exposure to green light without a prior adaptation period. This change in the light supply caused the lipid content to increase from 41.7 to 49.6% (Fig. 6b). However, the total lipid productivity remained unchanged, due to the reduction of the biomass production to 1.52 g L^−1^, which is 12.6% lower than the control without the light shift (Table 3).

**Table 3:** Total lipid content and biomass/lipid productivities of *N. gaditana* with/without shifting fluorescent dye

Experiment	Control	Shift
Biomass (g L^−1^)	1.708 ± 0.062	1.517 ± 0.332
Lipid content (%, g g biomass^−1^)	41.675 ± 3.638	49.644 ± 0.130*
Biomass productivity (g L^−1^ day^−1^)	0.259 ± 0.012	0.233 ± 0.060
Lipid productivity (g L^−1^ day^−1^)	0.108 ± 0.008	0.104 ± 0.030

Control indicates dye maintained with R101, and shift indicates dye having shifted at day 5. All measurements were achieved at the 6th day of cultivation. The data points represent the average of samples and error bars indicate standard error (*n* = 3). Significant differences, as determined by Student’s *t* test, are indicated by asterisks (* *P* < 0.05)

## Discussion

The three different fluorescent dyes tested here were found to have different characteristic wavelengths with substantial conversion of actual incident solar radiation, and as a result they were hypothesized to exhibit the following effects on the growth of *N. gaditana*. Since the absorption and emission spectra of a dye overlap in certain wavelength range, the actual conversion of solar radiation reflected on both the absorption and emission characteristics of each dye. Chl *a* has peak absorption wavelengths at blue and red colors, specifically between 440 and 500 nm and 660–700 nm. The shorter blue wavelength almost exactly overlaps the range that is intensified by DPA (Fig. 1c). Photons in the range of UV, which are outside the PAR of microalgae, were absorbed using DPA and were re-emitted in the blue range. This had the simultaneous benefit of increasing the distribution of the incident light in the PAR range and at the same time reducing the detrimental UV wavelength, both of which led to enhanced growth. The DPA-modified light consists of a greater portion of blue wavelength, which could be utilized by chl *a*, a major pigment of *N. gaditana.* A number of previous studies reported the beneficial effects of blue light on microalgal growth, where Okumura et al. used blue LED (emission range in between 450 and 500 nm) and Vadiveloo et al. used halogen lamps covering 400 and 525 nm [11, 12].

Although green light does not have any harmful effects, unlike UV, it cannot be used by strains of *Nannochloropsis*. Consequently, R101, which converts green light to red light that is usable by chl *a*, was also expected to have a positive impact on the growth of *N. gaditana*. Previous studies on *Nannochloropsis* reported growth promoting effects that are similar to our study, where Schulze et al. and Kim et al. demonstrated the effectiveness of monochromatic red LEDs (630–690 nm) as light source [10, 17].

When compared to a previous report on the use of DPA and R101, it is evident that both fluorescent dyes are effective for promoting growth of microalgae [18]. The growth of *Chlorella vulgaris* was maximized with the largest photosynthetic efficiency being achieved with R101, while DPA also promoted the growth up to 1.4 g L^−1^. Although *C. vulgaris* and *N. gaditana* differ in terms of photosynthetic pigment composition, both possess chl *a* as a main pigment which utilizes blue and red wavelengths. Thus, the growth promoting effect that was observed in both strains when they were subjected under DPA- and R101-modified lights strongly indicates the enhanced photosynthetic efficiency.

R110, which converts blue light to green light, was anticipated to impair growth. The reduction of the blue region appeared to negatively affect the growth, at least in the early stage. Interestingly, the cells were able to adapt to this unfavorable light condition by the exponential phase (on 3 days of cultivation) and resume normal growth behavior.

The varied responses to different wavelength could be explained to a certain degree in terms of photosynthetic characteristics. In phototrophic cultivation, growth depends entirely on cells’ photosynthesis capacity. This is due to the fact that ATP and NADPH, both of which are energy carriers for CO_2_ fixation and macromolecule synthesis, are generated from the process [2, 3, 19]. Since the light energy is harnessed through pigments such as chlorophylls, which have a specific absorption spectrum, the availability and extent of wavelength of incident light play a key part in the activity as well.

The degree of photosynthesis is commonly expressed in terms of maximal quantum yield (*F*_v_/*F*_m_), a parameter representing the maximal ability of photochemical energy conversion [20]. This parameter is a practical way of measuring the activity of photosystem II (PS II).

The *F*_v_/*F*_m_ values in Fig. 3 reflected the growth pattern in a proportional fashion; the differences in *F*_v_/*F*_m_ values between DPA/R101 and R110/control were reflected by the growth profile (Fig. 2). It is also noteworthy that the *F*_v_/*F*_m_ values increased for R110- and ethanol-modified lights at day 6. The cells divided exponentially for both cases, which indicates that photosynthesis was active under these conditions. On the contrary, *N. gaditana* cells exposed to DPA- or R101-modified light were near the stationary growth phase at day 6 and apparently ceased to divide at this particular time. The decrease in *F*_v_/*F*_m_ was substantial, especially for DPA-modified light, which shows that photosynthesis was not very active with blue-strengthened light at this point of cultivation. It was expected that photosynthetic activity would drop on day 10 (late stationary growth phase) across all conditions due to nutrient depletion. When a nitrogen source is not sufficiently supplied, *F*_v_/*F*_m_ values tend to decrease. In the case of *N. gaditana*, the *F*_v_/*F*_m_ values can drop below 0.5, which indicates ill status [21]. In the present study, the values on day 10 were low at 0.526 for R110-modified light and 0.514 for the control, which correspond to 14.9 and 13.7% decreases compared to the values at day 6, respectively. This did not occur in cultures under DPA- and R101-modified light, although cells were also similarly at the late stationary growth phase (Fig. 2). In both cultures, the cell division stopped even from day 6 of cultivation, which was confirmed by decreased *F*_v_/*F*_m_ values at day 6. Relatively high values of *F*_v_/*F*_m_ in cultures under DPA- and R101-modified light at day 10 could be related to the increasing tendency of dry cell weight in Fig. 2b. The apparent cessation of cell division and accumulation of biomass suggest that the photochemical conversion of light energy at this point of cultivation resulted in the synthesis of storage compounds through the alteration of carbon flux [19]. Changes in pigment composition also appeared to play a role in the whole growth pattern of *N. gaditana* with respect to light wavelength. This is because *Nannochloropsis* possesses chl *a* and xanthophylls as major photosynthetic pigments, and the two have different roles [9]. Chl *a*, the most abundant of all, absorbs mainly blue and red wavelengths and accounts for most of the photochemical conversion. Carotenoids, which play a minor part in light harvesting in *Nannochloropsis*, absorb green to orange wavelengths. Their primary role is photoprotection, which is an important mechanism when microalgae are exposed to harmful light conditions and damaged by photoinhibition [22]. When excess amount of light energy is supplied to a cell, chl *a* is overexcited to triplet state. Then, it transfers its excess energy to molecular oxygen, which causes the formation of harmful reactive oxygen species (ROS). Carotenoids can protect the cells from photoinhibition by preventing the formation of overexcited chl *a*, quenching the energy of excited chl *a*, and scavenging the ROS for removal. In *Nannochloropsis*, main carotenoids are beta-carotene and xanthophyll such as violaxanthin and zeaxanthin [9]. Beta-carotene is formed from geranyl–geranyl-PP which is synthesized from light, CO_2_ and water. Zeaxanthin and violaxanthin are part of violaxanthin cycle, which branches from beta-carotene production. Amount of pigments are adjusted as according to the roles they perform in terms of photoprotection, where beta-carotene participates in filtering, quenching and scavenging, while zeaxanthin and violaxanthin are only known to be involved in quenching and scavenging mechanisms [22].

Daily changing patterns in contents of chl *a* for each light conditions were well-correlated to *F*_v_/*F*_m_ values (Fig. 3), implying that the increased portion of chl *a* is related with elevated photochemical conversion yield. For DPA and R101, both *F*_v_/*F*_m_ as well as chl *a* values decreased consistently from day 2 to 10. Meanwhile, the control and R110 showed a pattern where both *F*_v_/*F*_m_ and chl *a* spiked on day 6 before falling to the lowest values on day 10. This was solidly supported by Figs. 3, 4, which shows that chl *a* had close relationships with photochemical conversion activity in PS II, and thus with the growth of *N. gaditana*. All this was rather anticipated, as the major role of chl *a* is to aid photochemical conversion during the light harvesting process, which in turn facilitates the generation of ATP and NADPH [19, 22].

Another noticeable observation is the changes in the carotenoid contents. These pigments are involved in the cells’ photoprotective mechanisms, and hence their abnormal levels are indicative of suboptimal growth [22]. Cell growth was additionally inhibited at the 2nd day of cultivation in the case of R110-modified light (Fig. 2), due to the absence of blue range. Under this suboptimal light condition, chl *a* content was found to be relatively lower and violaxanthin and beta-carotene were comparatively higher as a result (Fig. 4d). Also, at day 10, in which cells were in the late stationary phase, chl *a* content was reduced to below 2% of biomass for all conditions (Fig. 4c). At this stage, beta-carotene became the predominant pigment, which is a natural consequence of nutrient starvation (Fig. 4f).

Lipid productivity is the most important parameter with respect to microalgae-based biofuel production [23]. It is determined by a combination of cell growth rate and lipid content inside the cell body, and thus both the growth rate and lipid content are important. When cell division and growth cease, carbon and energy flux are changed in direction towards synthesis of storage materials. This results in a compositional redistribution among proteins, carbohydrates, and lipids [19, 24]. A desired rise in lipid content coincides with reduction in protein and carbohydrate content. In every light condition, total lipid contents were over 50% of dry biomass (Fig. 5). Considering that the general lipid content of *Nannochloropsis* spp. is around 30%, nitrogen deprivation was likely in all the cases [25]. Unlike our expectation, the dye-based light conversion had no significant effect on biochemical composition after 10 days of cultivation (Fig. 5).

This result is counterintuitive, because growth enhancement is generally achieved at the expense of lipid content [26]. Also, the result is contradictory to the previous report using DPA- and R101-modified light, which mentioned that the total lipid content was elevated up to 30% in *C. vulgaris* [18]. It appeared that the biochemical compositions at day 10 were mostly influenced by nitrogen deprivation, instead of being affected by specific wavelength. The adaptability of *N. gaditana* to different wavelength compositions could be another reason why the wavelength conversion had no clear effect, as shown with R110-modified light in Fig. 2. As a consequence, the lipid productivities calculated after 10 days of cultivation were solely influenced by variations of biomass accumulation, not by lipid content (Table 2). Previous studies using a single light color illumination—Kim et al. with monochromatic red LED and Teo et al. with blue (457 nm) LED reported contradictory results [17, 27]. In fact, whether monochromatic wavelength has any effect on lipid content is still controversial, since the exact mechanism of harvested light on biosynthesis of protein, carbohydrate, and lipid macromolecules has not yet been clarified.

Sudden and vast accumulation of lipids is expected when dramatic changes in the external environment are provided to the cells, such as nitrogen starvation or high light intensity [28]. If enough time for adaptation is not given to cells, the lipid content would show noticeable differences according to incident wavelength changes. With R101-modified light, green wavelength was absent for 5 days initially, before the cells were exposed to an excess of green light through the sudden shift of fluorescent dye to R110. This shift resulted in a decrease of desired wavelength and an increase of undesired wavelength in terms of chl *a*, which is a major pigment for photochemical conversion. Since both blue and red colors are the most favored spectra of chl *a*, it inevitably results in a decrease of photosynthetic activity, which results in a reduction of cell division (Fig. 6a). Although there is no known molecular mechanism that can explain the relationship between green wavelength and lipid biosynthesis, subjecting the cells under green LED (520 nm) was previously shown to be effective for lipid induction in *Nannochloropsis* [29]. In this study, *N. gaditana* also showed additional accumulation of lipids when the fluorescent dye was switched in the middle of cultivation to R110 (Fig. 6b), supporting that the abrupt introduction of changes in wavelength composition was more effective than supplying an undesired wavelength for an extended period of time.

This seemingly useful strategy of lipid induction, however, turned out to be not very effective at improving the total lipid productivity, because the increase in the lipid content took place at the cost of growth (Table 3). Even so, the dye-based manner of stimulating lipid production could still have potential for utilization in continuous mode of cultivation, such as a system based on a continuous multi-stage process [30].

## Conclusions

Conversion of unused and/or harmful portions of incident sunlight into usable photons using fluorescent dyes was found to be an effective approach for improving the microalgal photosynthetic output. Both biomass and lipid productivities of *N. gaditana* increased by 40% under this approach. A lipid induction effect was verified when changing fluorescent dye from R101 (red) to R110 (green) during cultivation, which resulted in an induction of 19.1% of additional lipid. These results suggest that changing the composition of sunlight wavelength using fluorescent dyes can be utilized widely in the field of microalgal cultivation.

## Methods

### Fluorescent dyes

Three different fluorescent dyes were used to convert the spectrum of incident sunlight. To convert ultraviolet (UV) spectra to the visible range, 9,10-diphenylanthracene (DPA) was used. Rhodamine 110 (R110) was used to absorb blue spectra and emit green range. Finally, rhodamine 101 (R101) was used to convert green range to red. All fluorescent dyes were purchased from Sigma-Aldrich Co. To measure absorption and emission characteristics of each dye, they were dissolved in ethanol with concentration of 10^−5^ M. Absorption spectra of dyes were measured using a UV-Vis spectrophotometer (Shimadzu Co., Japan) using ethanol as a baseline. The absorption spectrum of *N. gaditana* was also measured with the same device, using distilled water as a baseline. Emission spectra of dyes were measured using a spectrofluorophotometer (Shimadzu Co., Japan) with an excitation wavelength at 620 nm. Spectral distribution of original light source and dye-modified lights were measured using a spectrometer (Soma Co., Japan). Every spectrum was investigated from 300 to 800 nm, and normalized to each peak wavelength.

### Culture conditions and photobioreactor set-up

The seawater strain *Nannochloropsis gaditana* CCMP526 (National Center for Marine Algae and Microbiota, Maine, USA) was cultivated in this study. A culture of *N. gaditana* was maintained in a sterile modified f/2 medium [31], with the following composition: 30 g L^−1^ sea salts, 375 mg L^−1^ NaNO_3_, 25 mg L^−1^ NaH_2_PO_4_∙9H_2_O, 15.75 mg L^−1^ FeCl_3_∙6H_2_O, 21.80 mg L^−1^ Na_2_EDTA∙2H_2_O, 49.0 μg L^−1^ CuSO_4_∙5H_2_O, 31.5 μg L^−1^ Na_2_MoO_4_∙2H_2_O, 110 μg L^−1^ ZnSO_4_∙7H_2_O, 50 μg L^−1^ CoCl_2_∙6H_2_O, 900 μg L^−1^ MnCl_2_∙4H_2_O, 2.5 μg L^−1^ vitamin B_12_, 2.5 μg L^−1^ biotin, and 500 μg L^−1^ thiamine hydrochloride. The culture was retained in a culture flask with 300 ml working volume, 200 μmol photons m^−2^ s^−1^ of white LED light and 25 °C.

A flat-panel photobioreactor (flat-panel PBR) made of polyvinyl chloride (PVC) body frames was used in the present study. The PBR was composed of width, height, and thickness of 220, 335, and 30 mm, respectively, and the total working volume was 2.25 L. A solar simulator (McScience Co., Republic of Korea) having a spectral composition corresponding to the sun, was used to illuminate 200 μmol photons m^−2^ s^−1^ of light intensity to the PBR surface for all experimental conditions. The surface of PBR was composed of transparent indium tin oxide (ITO) glass with a light path of 30 mm. A layer containing 10^−5^ M fluorescent dyes dissolved in ethanol was installed ahead of the main volume of PBR with a thickness of 20 mm. The light passing through the dye layer was then illuminated to culture of *N. gaditana* (Additional file 1: Figure S1). Air mixed with CO_2_ (3%, v/v) was supplied through a polyurethane tube located at the bottom of PBR and the gas bubbles generated mixing in every volume of PBR. The culture was kept at 25 °C using a cooling chamber that circulated water at the back side of PBR. All cultivations were implemented in biological triplicate.

### Growth measurement

The cell density of *N. gaditana* was determined using a Cellometer Auto X4 Cell Counter (Nexcelom Bioscience, USA). Samples were injected into a cell counting chamber with 20 μL volume each and measured in duplicate.

For measurement of the dry cell weight, cellulose nitrate membrane filters with a 0.45 μm pore size (Whatman, USA) were pre-dried in an oven at 80 °C. Suspensions of *N. gaditana* were passed through the filters using a vacuum pump, and then the filters were dried at 80 °C overnight. The dry cell weight was determined as follows:

$${\text{Dry cell weight}} = \frac{{\left( {\text{weight after filtration}} \right) - ({\text{weight before filtration}})}}{\text{volume of filtration}}$$

Specific growth rate (*μ*) was calculated as follows:

$$\mu = \frac{{\ln X_{2} - \ln X_{1} }}{{t_{2} - t_{1} }}$$

where *t* is time and *X*_*i*_ is the dry cell weight of *N. gaditana* at time *t*_*i*_.

### Chlorophyll fluorescence measurement

Photosynthetic characteristics of *N. gaditana* at different incident wavelength profiles were investigated using multi-color pulse–amplitude modulation (Multi-PAM; Heinz-Walz, Germany). The wavelength-dependent characteristics of fluorescence quantum yield were measured following previously described methods [20, 32]. Briefly, *N. gaditana* cultures were diluted to 300 μg chlorophyll *a* L^−1^ with modified f/2 medium, and then dark-adapted for 20 min. The dark-adapted samples were measured at 440 nm using the SP-analysis mode. The *F*_v_/*F*_m_ values at each wavelength were calculated and presented automatically.

### Analytical methods

Content and composition of photosynthetic pigments were investigated using High Performance Liquid Chromatography (HPLC, Dionex Ultimate 3000, Thermo Fisher Scientific, USA) with l Dionex Acclaim™ 120 5 μm C18 column (Thermo Fisher Scientific, USA). A UV–visible detector (Ultimate 3000 VWD Variable Wavelength Detector, Thermo Fisher Scientific, USA) was used at 440 nm. The column oven temperature of the HPLC was maintained at 25 °C. Two eluents (methanol:0.5 M ammonium acetate = 8:2 (v/v), methanol: acetone = 7:3 (v/v)) were supplied at a flow rate of 0.8 mL min^−1^ for analysis. The ratio of two eluents flowing into the column gradually changed at a pre-set time interval. Each peak of the chromatograph was compared with those of chlorophyll *a*, beta-carotene, violaxanthin, and zeaxanthin (Sigma-aldrich, USA), and each standard was prepared with a concentration gradient of 0.2/1/5/10/50 μg mL^−1^. Concentrations of each pigment were acquired using a calibration curve with individual standards.

Lipid content was analyzed using the modified Folch method [33]. Suspensions of *N. gaditana* were harvested and centrifuged at 7000 rpm for 10 min. The harvested pellets were washed twice using distilled water and then stored at − 70 °C for 24 h. The frozen cells were freeze-dried (Ilshin, Republic of Korea) for 4 days and then ground into fine powders. They were treated with a solution of chloroform/methanol (2:1, v/v) to extract lipids. Phase separation was achieved by adding water, followed by centrifugation at 4000 rpm for 10 min. The lower phase was handled with a syringe, and left overnight to evaporate remaining solvents. The total lipid content was calculated as follows:

$$ {\text{Total lipid content }}\left( {{\text{\%}}, {\text{g oil g biomass}}^{ - 1} } \right) = \frac{{(W_{\text{L}} - W_{\text{D}} ) \times V_{\text{C}} }}{{V_{\text{P}} \times W_{\text{S}} }} \times 100 $$

where *W*_L_ is the weight of the dish containing the lipid sample, *W*_D_ is the weight of the dish, *W*_S_ is the weight of dry biomass, *V*_C_ is the added volume of chloroform, and *V*_P_ is the volume of chloroform moved to the dish.

Carbohydrate content was obtained using a phenol–sulfuric acid assay [34]. Dry biomass was dissolved in distilled water and treated with 5% phenol (dissolved in distilled water) and concentrated sulfuric acid. Reaction was taken for 30 min, and then the absorbance of the sample was measured at 470 nm. The carbohydrate concentration was calculated using a calibration curve achieved by known concentrations of pure glucose dissolved in distilled water.

To measure ash content in dry biomass, an aluminum dish was pre-burned in a furnace at 575 °C for 3 h and then the weight of the burned dish was measured. The weight of the dry biomass sample was measured afterwards, and then the dish containing the biomass was burned in the furnace at the same conditions mentioned above. The final ash content was calculated as follows:

$${\text{Ash content }}\left( {{\text{\%}}, {\text{g g biomass}}^{ - 1} } \right) = \frac{{W_{3} - W_{1} }}{{W_{2} - W_{1} }} \times 100$$

where *W*_1_ is the weight of the burned dish, *W*_2_ is the weight of the dish with dry biomass, and *W*_3_ is the weight of the burned dish containing burned biomass.

Protein content was presented as follows:

$${\text{Protein content }}\left( {{\text{\%}}, {\text{g g biomass}}^{ - 1} } \right) = 100 - C_{\text{lipid}} - C_{\text{carbohydrate}} - C_{\text{ash}}$$

where *C*_*i*_ is the content of *i* in dry biomass.

## Additional file

**Additional file 1: Figure S1.** Configuration of PBR with dye-filled layer.

## Abbreviations

*N. gaditana*: *Nannochloropsis gaditana*
PAR: photosynthetic active radiation
LED: light-emitting diode
DPA: 9,10-diphenylanthracene
R110: rhodamine 110
R101: rhodamine 101
PBR: photobioreactor
*F*_v_/*F*_m_: maximal quantum yield of photosystem II
PAM: pulse–amplitude modulation
Chl: chlorophyll
UV: ultraviolet
PS II: photosystem II
*C. vulgaris*: *Chlorella vulgaris*
