# Supplementary figures and images for: Wavelength shift strategy to enhance lipid productivity of Nannochloropsis gaditana
Source: Biotechnol Biofuels. 2018 Mar 19;11:70. doi: 10.1186/s13068-018-1067-2 (PMC5858150; doi:10.1186/s13068-018-1067-2)

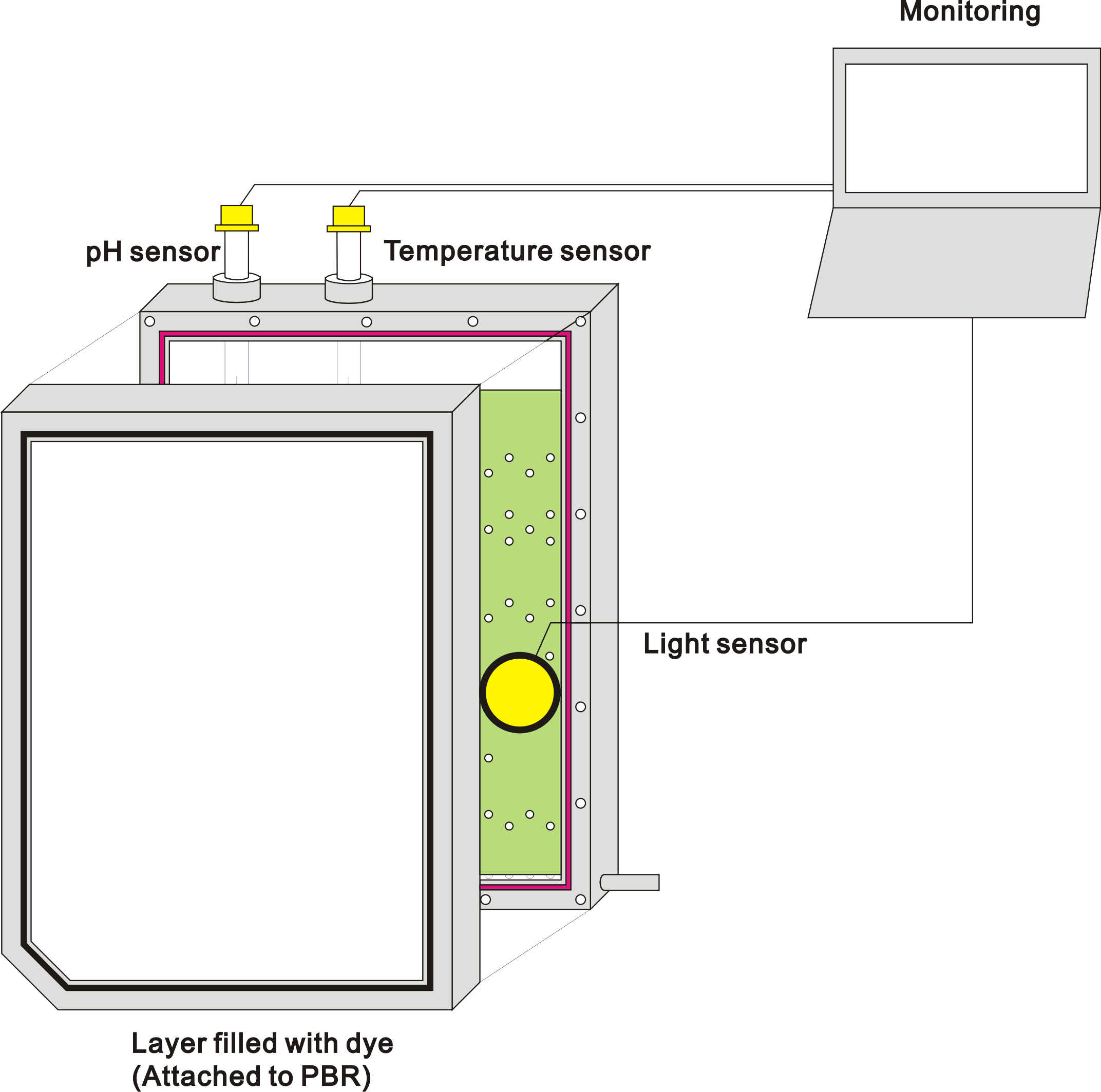

Supplement: Supplementary file 1 — Additional file 1: Figure S1. Configuration of PBR with dye-filled layer. [file 13068_2018_1067_MOESM1_ESM.tif]
